# Challenges in evaluating treatments for COVID-19: The case of in-hospital anticoagulant use and the risk of adverse outcomes

**DOI:** 10.3389/fphar.2022.1034636

**Published:** 2022-11-24

**Authors:** Ya-Hui Yu, In-Sun Oh, Han Eol Jeong, Robert W. Platt, Antonios Douros, Ju-Young Shin, Kristian B. Filion

**Affiliations:** ^1^ Department of Epidemiology, Biostatistics and Occupational Health, McGill University, Montreal, QC, Canada; ^2^ Centre for Clinical Epidemiology, Lady Davis Institute, Jewish General Hospital, Montreal, QC, Canada; ^3^ School of Pharmacy Science, Sungkyunkwan University, Suwon, Gyeonggi-do, South Korea; ^4^ Department of Biohealth Regulatory Science, Sungkyunkwan University, Suwon, Gyeonggi-do, South Kore; ^5^ Department of Pediatrics, McGill University, Montreal, QC, Canada; ^6^ Department of Medicine, McGill University, Montreal, QC, Canada; ^7^ Institute of Clinical Pharmacology and Toxicology, Charité-Universitätsmedizin Berlin, Berlin, Germany; ^8^ Department of Clinical Research Design and Evaluation, Samsung Advanced Institute for Health Sciences and Technology, Seoul, South Korea

**Keywords:** anticoagulants, COVID-19, mortality, South Korea, observational study

## Abstract

Anticoagulants are a potential treatment for the thrombotic complications resulting from COVID-19. We aimed to determine the association between anticoagulant use and adverse outcomes among hospitalized patients with COVID-19. We used data from the COVID-19 International Collaborative Research Project in South Korea from January to June 2020. We defined exposure using an intention-to-treat approach, with person-time classified as use or non-use of anticoagulants at cohort entry, and a time-varying approach. The primary outcome was all-cause, in-hospital mortality; the secondary outcome was a composite including respiratory outcomes, cardiovascular outcomes, venous thromboembolism, major bleeding, and intensive care unit admission. Cox proportional hazards models estimated adjusted hazard ratios (HRs) of the outcomes comparing use versus non-use of anticoagulants. Our cohort included 2,677 hospitalized COVID-19 patients, of whom 24 received anticoagulants at cohort entry. Users were older and had more comorbidities. The crude incidence rate (per 1,000 person-days) of mortality was 5.83 (95% CI: 2.80, 10.72) among anticoagulant users and 1.36 (95% CI: 1.14, 1.59) for non-users. Crude rates of the composite outcome were 3.20 (95% CI: 1.04, 7.47) and 1.80 (95% CI: 1.54, 2.08), respectively. Adjusted HRs for mortality (HR: 1.12, 95% CI: 0.48, 2.64) and the composite outcome (HR: 0.79, 95% CI: 0.28, 2.18) were inconclusive. Although our study was not able to draw conclusions on anticoagulant effectiveness for COVID-19 outcomes, these results can contribute to future knowledge syntheses of this important question. Our study demonstrated that the dynamic pandemic environment may have important implications for observational studies of COVID-19 treatment effectiveness.

## Introduction

The coronavirus disease 2019 (COVID-19), caused by severe acute respiratory syndrome coronavirus (SARS-CoV-2) infection, has resulted in over five million deaths worldwide (WHO, 2022). Research has centered on identifying existing therapies that can be repurposed to prevent the progression of COVID-19 and death (Khani et al., 2021). Evidence evaluating the effect of treatments on COVID-19 comes mainly from observational studies due to the limited understanding of the disease at early pandemic stages and the longer time required to conduct clinical trials. Determining the causal effects of treatment from observational studies is inherently challenging, and the rapidly evolving pandemic environment poses additional design and analytical hurdles (Griffith et al., 2020; Pottegård et al., 2020), leading to biased results in the literature (Cohen et al., 2021; Renoux et al., 2021).

Anticoagulants have been suggested as a potential treatment for the thrombotic complications resulting from COVID-19, since coagulopathy has emerged as an important clinical feature of the disease (Iba et al., 2020; Lippi et al., 2021). A high incidence of venous thromboembolism (VTE) has also been observed among patients hospitalized with COVID-19, with a pooled estimate of 17% (Jiménez et al., 2021). Although most COVID-19 studies of anticoagulants have shown improved survival, important heterogeneity exists between these studies (e.g., different timing of initiation, dose) (Albani et al., 2020; Ayerbe et al., 2020; Billett et al., 2020; Lynn et al., 2020; Nadkarni et al., 2020; Paranjpe et al., 2020; Tang et al., 2020; Di Castelnuovo et al., 2021; Hara et al., 2021; Ionescu et al., 2021; Rentsch et al., 2021). These studies were also subject to important methodological limitations; for example, the grouping of patients based on anticoagulant use throughout follow-up may have induced immortal-time bias in several studies (Albani et al., 2020; Lynn et al., 2020; Paranjpe et al., 2020; Tang et al., 2020; Di Castelnuovo et al., 2021; Hara et al., 2021; Ionescu et al., 2021). In addition, most were single-center studies or had small sample sizes. Currently, few studies (Hara et al., 2021; Rentsch et al., 2021) used large population-based cohorts; while one study (Rentsch et al., 2021) used U.S. Veterans Affairs data focused on the impact of early anticoagulation initiation and did not assess the impact of time-varying anticoagulant use.

Our objective was to determine the association between anticoagulant use and the risk of adverse outcomes among hospitalized patients with COVID-19 in a population-based nationwide insurance database in South Korea. Specifically, we examined the association between the inpatient anticoagulant use and the risk of all-cause, in-hospital mortality as well as the risk of a composite outcome that included respiratory outcomes, cardiovascular outcomes, VTE, major bleeding, and intensive care unit (ICU) admission.

## Methods

### Hypothetical target trial

The target trial framework has been recognized as a useful tool in guiding observational study design to emulate a trial that would have been performed in an ideal scenario (Hernán and Robins, 2016). This framework has been applied widely to various research questions (e.g., the association between corticosteroids and the risk of COVID-19 mortality (Hoffman et al., 2022)). The components of the hypothetical target trial in our study are listed in Supplementary Appendix SA1. Briefly, this target trial aimed to compare the risk of in-hospital mortality among patients who use anticoagulants during their entire hospital stay due to COVID-19 versus those who did not use an anticoagulant. The causal contrasts of interest were intention-to-treat and per-protocol effects. The following sections describe the details of how we emulated this hypothetical target trial using data from a population-based cohort in South Korea.

### Data source

We used data from the COVID-19 International Collaborative Research Project, a South Korean initiative developed to provide real-world data for COVID-19 research (Rho et al., 2021). All patients with COVID-19 were identified through insurance claims from the National Health Insurance (NHI) system of Korea by Health Insurance Review and Assessment Service. In South Korea, all residents (Korean nationals and foreigners) are covered by the NHI system operated by the Korean government as a single payer. Residents are enrolled in this system from birth until emigration or death. This nationwide claim database provides longitudinal information on an individual-level for sociodemographic factors and all healthcare-related services (including prescription medications) from inpatient, outpatient, nursing home settings, and from all levels of care (Kim et al., 2017; Kyoung and Kim, 2022). This COVID-19 database was established and linked to the nationwide claim database which contains information regarding inpatient and outpatient diagnoses, procedures, and prescription drugs, as well as patients’ most recent 3-year history of medical services.

Diagnoses and procedures were classified using the International Classification of Diseases, 10th Revision, Clinical Modification (ICD-10-CM) codes; drug prescriptions were classified using a domestic coding system corresponding to the World Health Organization-Anatomical Therapeutic Chemical Classification (WHO-ATC). The Research Ethics Boards of Sungkyunkwan University (Suwon, South Korea) and of Jewish General Hospital (Montreal, Canada) approved this study.

### Study population and follow-up

We included all patients with COVID-19-related hospitalizations between 20 January and 4 June, 2020. COVID-19 was identified using domestic codes reflecting a recorded positive test result. During this period, diagnostic criteria for COVID-19 in South Korea were based on the result from the reverse transcription polymerase chain reaction test kits, which were approved by the Korean Ministry of Food and Drug Safety (WHO, 2020). All the residents of South Korea (of all nationalities) had access to PCR COVID-19 tests at the designated test centers closest to their residence without additional costs regardless of their symptoms.

The cohort entry date was defined by the date of hospital admission or the date of COVID-19 diagnosis during hospitalization, whichever occurred last. All patients were followed from the cohort entry date until an event or censoring due to hospital discharge, in-hospital death (for outcomes other than mortality), or the end of the study period, whichever occurred first. When a patient had multiple COVID-19-related hospitalizations, we only included the first.

We excluded patients aged less than 20 years at cohort entry. We also excluded those with a cancer diagnosis or recorded cancer-related treatments in the year before cohort entry (other than non-melanoma skin cancer). Patients who were newly prescribed an anticoagulant and had a diagnosis of the outcome recorded on the same date were excluded. We excluded these patients as we could not establish the temporal order of exposure and outcome (i.e., that patients initiated anticoagulants prior to the outcome occurrence) based on their reimbursement claims. The graphic presentation of the time windows for cohort entry and assessing exclusion criteria, exposure, outcome, and potential confounders can be found in Supplementary Figure S1.

### Exposure definitions

In-hospital medication use was ascertained based on the prescription claims including information on date of prescription, days of supply, dose, route of administration, etc. These claims included both inpatient and outpatient claims. We first defined exposure using an intention-to-treat (ITT) approach, with person-time classified as either anticoagulant use or non-use based on reception at cohort entry. To account for the dynamic nature of anticoagulant use, we also used a time-varying exposure where each person-day was classified: 1) current use of anticoagulants; or 2) no current use of anticoagulants. In the time-varying approach, exposure status was updated daily with current use defined by a prescription for any anticoagulant on the day for which the exposure was being defined. Anticoagulants included vitamin K antagonists, direct oral anticoagulants, heparin [low molecular weight heparin (LMWH) or unfractionated heparin (UFH)], and fondaparinux.

### Outcome definitions

The primary outcome of this study was in-hospital, all-cause mortality. The secondary outcomes were a composite outcome of respiratory outcomes, cardiovascular outcomes, VTE, major bleeding and ICU admission (Supplementary code list S1), and the individual components of this composite endpoint. The ICD-10-CM codes for primary and secondary diagnoses were used to identify these outcomes (Supplementary code list). When multiple records identified the same outcome, the earliest recorded date defined the event date. For composite outcomes, the date of the earliest event defined the event date.

### Potential confounders

We identified patient demographic and clinical characteristics that may be associated with anticoagulant use or the risk of the outcomes of interest based on published evidence. Characteristics measured at cohort entry included demographics, calendar time of cohort entry, comorbidities and medication use (Supplementary code list S2). We assessed comorbidities using recorded diagnoses in the 3 years before cohort entry, and medications using recorded dispensing in the year before cohort entry. We included a variable to indicate major general surgery in the year before cohort entry, since those with recent surgeries were more likely to receive thrombosis prophylaxis (Nemeth et al., 2019). Two proxies for overall health in the prior year were included: number of unique medication classes dispensed and the number of hospitalizations. Comorbidities, medication use, major general surgery, and arising conditions (sepsis, disseminated intravascular coagulation, thrombocytopenia), ICU admission, and mechanical ventilator use were also assessed using a time-varying approach, with comorbidities updated daily and defined as having ever had a relevant diagnosis. Medication use was also updated daily. The code lists for defining the above potential confounders were provided in the Supplementary code list.

### Statistical analysis

We compared the characteristics of patients who received anticoagulants versus those who did not at cohort entry. Absolute standardized differences were computed for the difference between the treatment groups, and results greater than 0.1 were considered important (Austin, 2009). For the comparison of time-varying covariates, we reported the proportion of person-days of follow-up during which the covariate was present among person-days of current anticoagulant use versus no current use during hospitalization. Crude incidence rates and 95% confidence intervals (CIs) for each outcome of interest were calculated assuming Poisson distributions. Kaplan-Meier curves illustrated crude cumulative incidence proportions of each outcome by treatment group over follow-up.

For estimating the ITT effect, we used Cox proportional hazard models with follow-up time as the underlying time axis to estimate hazard ratios (HRs) and 95% CIs for each outcome for anticoagulant use versus non-use at cohort entry (ITT exposure definition). Due to the small number of patients who received anticoagulants, we were not able to perform the planned analyses using a marginal structural Cox model to estimate the effect of use of anticoagulant use on the outcomes that account for the change of anticoagulant use status during follow-up and the impact of time-varying confounders (per-protocol effect in our study). Instead, we used time-dependent Cox proportional hazard models to estimate HRs and 95% CIs for each outcome for current use versus no current use of anticoagulants (time-varying exposure definition). All models were adjusted for baseline covariates only, with age, number of unique medications used, and number of hospitalizations modeled as continuous variables and all remaining variables modeled categorically. The statistical analysis plans for the originally planned analyses that were not feasible due to sample size are provided in Supplementary Appendix SA3.

## Results

**FIGURE 1:**
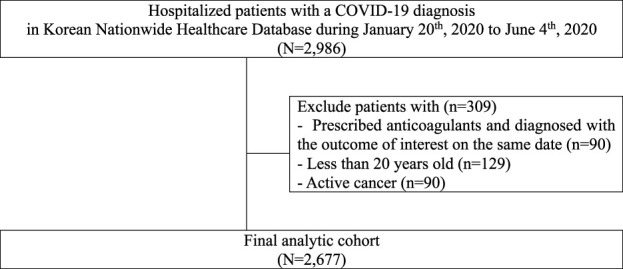
Flowchart describing patient selection for study of anticoagulant use among patients hospitalized with COVID-19 in South Korea.

Our cohort included 2,677 hospitalized patients with COVID-19 (Figure 1) with a total of 109,417 person-days follow-up time. The average follow-up time was 41 days, and the maximum follow-up time was 180 days. A total of 62.1% were aged 49 years or younger, and 52.7% were female. There were 156 all-cause in-hospital deaths and 181 composite adverse events.

A total of 24 patients received anticoagulants at cohort entry (13 received oral anticoagulants, eight parenteral anticoagulants, and 3 received both) (Supplementary Table S1). Compared to patients who did not receive anticoagulants (*n* = 2,653) at cohort entry, patients who received anticoagulants were more likely to be aged 70 + years, admitted in earlier months, obese, and have more comorbidities (Table 1). They also used more medications during the year before cohort entry, and 75% had a history of using anticoagulants. A total of 44 patients received anticoagulants at any time during hospitalization, and we observed similar trends in comorbidities and medications during hospitalization (Table 2). Prior to their current use of anticoagulants, patients were more likely to have comorbidities recorded or have used other medications including antidiabetics, antiplatelets, and statins during hospitalization compared to periods of non-use of anticoagulants.

**TABLE 1:** Baseline characteristics of COVID-19 hospitalized patients in South Korea between 20 January and 4 June, 2020, by use of anticoagulants at baseline.

	Total population (*n* = 2,677)		Non-use of anticoagulants (*n* = 2,653)		Use of anticoagulants (*n* = 24)		Standardized difference
Age (years)							2.09
20–29	981	(36.7)	981	(37.0)	0	(0.0)	
30–39	338	(12.6)	338	(12.7)	0	(0.0)	
40–49	343	(12.8)	341	(12.9)	2	(8.3)	
50–59	455	(17.0)	452	(17.0)	3	(12.5)	
60–69	261	(9.8)	260	(9.8)	1	(4.2)	
70–79	137	(5.1)	130	(4.9)	7	(29.2)	
80+	162	(6.1)	151	(5.7)	11	(45.8)	
Female	1,407	(52.6)	1,395	(52.6)	12	(50.0)	0.05
Calendar time of admission (tri-weekly)							1.37
20 January to 9 February, 2020	296	(11.1)	291	(11.0)	5	(20.8)	
10 February to 1 March, 2020	144	(5.4)	138	(5.2)	6	(25.0)	
2 March to 22 March, 2020	60	(2.2)	56	(2.1)	4	(16.7)	
23 March to 12 March, 2020	22	(0.8)	21	(0.8)	1	(4.2)	
13 April to 3 May, 2020	1,373	(51.3)	1,365	(51.5)	8	(33.3)	
4 May to 24 May, 2020	776	(29.0)	776	(29.3)	0	(0.0)	
24 May to 4 June, 2020	6	(0.2)	6	(0.2)	0	(0.0)	
Type of health insurance							0.05
Health insurance	2,414	(90.2)	2,392	(90.2)	22	(91.7)	
Medical aid	263	(9.8)	261	(9.8)	2	(8.3)	
Comorbidities^a^							
Obesity	30	(1.1)	16	(0.6)	14	(58.3)	1.64
Hypertension	283	(10.6)	276	(10.4)	7	(29.2)	0.49
Chronic kidney disease	15	(0.6)	15	(0.6)	0	(0.0)	0.12
Diabetes	135	(5.0)	129	(4.9)	6	(25.0)	0.59
Coronary artery disease	37	(1.4)	34	(1.3)	3	(12.5)	0.45
Cerebrovascular disease	60	(2.2)	50	(1.9)	10	(41.7)	1.10
Atrial fibrillation	1	(0.1)	1	(0.1)	0	(0.0)	0.03
Heart failure	197	(7.4)	188	(7.1)	9	(37.5)	0.79
Stroke	40	(1.5)	34	(1.3)	6	(25.0)	0.75
Myocardial infarction	332	(12.4)	315	(11.9)	17	(70.8)	1.50
Mechanical heart valves installation	1	(0.1)	0	(0.0)	1	(4.2)	0.30
Coronary revascularization	12	(0.5)	10	(0.4)	2	(8.3)	0.40
Venous thromboembolism	1	(0.1)	1	(0.1)	0	(0.0)	0.03
Cancer (other than non-melanoma skin)	88	(3.3)	86	(3.2)	2	(8.3)	0.22
Bleeding	128	(4.8)	120	(4.5)	8	(33.3)	0.79
Medication use^b^							
Anticoagulants	38	(1.4)	20	(0.8)	18	(75.0)	2.38
Antidiabetic drugs	155	(5.8)	148	(5.6)	7	(29.2)	0.66
Antihypertensive drugs^c^	22	(0.8)	22	(0.8)	0	(0.0)	0.13
Nonsteroidal anti-inflammatory drugs	1,021	(38.1)	1,010	(38.1)	11	(45.8)	0.16
Antiplatelets	168	(6.3)	160	(6.0)	8	(33.3)	0.73
Statins	135	(5.0)	126	(4.8)	9	(37.5)	0.88
Immunosuppressants	5	(0.2)	5	(0.2)	0	(0.0)	0.06
Antivirals	47	(1.8)	46	(1.7)	1	(4.2)	0.14
Corticosteroids	615	(23.0)	605	(22.8)	10	(41.7)	0.41
Remdesivir	0	(0.0)	0	(0.0)	0	(0.0)	0.00
Hormone replacement therapy	30	(1.1)	29	(1.1)	1	(4.2)	0.91
Testosterone	0	(0.0)	0	(0.0)	0	(0.0)	0.00
Tamoxifen	1	(0.0)	1	(0.1)	0	(0.0)	0.03
Selective serotonin reuptake inhibitors	79	(3.0)	79	(3.0)	0	(0.0)	0.25
Surgery^b^	517	(19.3)	507	(19.1)	10	(41.7)	0.51
Numbers of unique medications dispensed, median (IQR)^a^	5	(0, 12)	5	(0, 12)	11	(3, 17)	0.97
Numbers of hospitalizations, median (IQR)^a^	0	(0, 0)	0	(0, 0)	0	(0, 5)	0.35

^a^ Assessed within 1 year prior to the cohort entry.

^b^ Assessed within 3 years prior to the cohort entry.

^c^ Includes Antiadrenergic agents, agents acting on Arteriolar smooth muscle, and Other antihypertensives not classified elsewhere in the WHO ATC.

**TABLE 2:** Distributions of time-varying covariates measured during hospitalization of COVID-19 hospitalized patients in South Korea between 20 January and 4 June 2020, by person-time of current use versus non-current use of anticoagulants.

	Total follow-up^a^ (*n* = 109,417 person-days)		Non-current use of anticoagulants (*n* = 107,948 person-days)		Current use of anticoagulants (*n* = 1,469 person-days)		Standardized difference
Comorbidities							
Obesity	1,035	(1.0)	704	(0.7)	331	(22.5)	0.73
Hypertension	3,996	(3.7)	3,996	(3.7)	0	(0.00)	0.28
Chronic kidney disease	580	(0.5)	518	(0.5)	62	(4.2)	0.25
Diabetes	8,060	(7.4)	8,011	(7.4)	49	(3.3)	0.18
Coronary artery disease	613	(0.6)	613	(0.6)	0	(0.0)	0.11
Cerebrovascular disease	1,314	(1.2)	1,113	(1.0)	201	(13.7)	0.50
Atrial fibrillation	0	(0.0)	0	(0.0)	0	(0.0)	0.00
Heart failure	8,863	(8.1)	8,608	(8.0)	255	(17.4)	0.29
Stroke	703	(0.6)	675	(0.6)	28	(1.91)	0.12
Myocardial infarction	15,028	(13.7)	14,597	(13.5)	431	(29.3)	0.39
Mechanical heart valves installation	43	(0.1)	0	(0.0)	43	(2.9)	0.25
Coronary revascularization	364	(0.3)	364	(0.3)	0	(0.0)	0.08
Venous thromboembolism	0	(0.0)	0	(0.0)	0	(0.0)	0.00
Cancer (other than non-melanoma skin)	5,215	(4.8)	5,176	(4.8)	39	(2.7)	0.11
Bleeding	5,978	(5.5)	5,911	(5.5)	67	(4.6)	0.04
Sepsis	182	(0.2)	182	(0.2)	0	(0.0)	0.06
Disseminated intravascular coagulation	11	(0.01)	11	(0.01)	0	(0.0)	0.02
Thrombocytopenia	105	(0.1)	105	(0.1)	0	(0.0)	0.05
Medication use							
Antidiabetic drugs	14,316	(13.1)	13,803	(12.8)	513	(34.9)	0.54
Antihypertensive drugs	2,778	(2.5)	2,768	(2.6)	10	(0.7)	0.15
Nonsteroidal anti-inflammatory drugs	33,504	(30.6)	33,007	(30.6)	497	(33.8)	0.07
Antiplatelets	12,302	(11.2)	11,845	(11.0)	457	(31.1)	0.51
Statins	10,252	(9.4)	9,717	(9.0)	535	(36.4)	0.69
Immunosuppressants	78	(0.1)	78	(0.1)	0	(0.0)	0.04
Antivirals	27,416	(25.1)	27,102	(25.1)	314	(21.4)	0.09
Corticosteroids	16,705	(15.3)	16,355	(15.2)	350	(23.8)	0.22
Remdesivir	0	(0.0)	0	(0.0)	0	(0.0)	0.00
Hormone replacement therapy	545	(0.5)	545	(0.5)	0	(0.0)	0.10
Testosterone	0	(0.0)	0	(0.0)	0	(0.0)	0.00
Tamoxifen	0	(0.0)	0	(0.0)	0	(0.0)	0.00
Selective serotonin reuptake inhibitors	4,965	(4.5)	4,963	(4.6)	2	(0.5)	0.29
Surgery	30,737	(28.1)	30,208	(28.0)	529	(36.0)	0.17
ICU admission	3,537	(3.2)	3,519	(3.3)	18	(1.2)	0.14
Ventilator use	792	(0.7)	792	(0.7)	0	(0.0)	0.12

^a^ Based on the follow-up time of the primary outcome.

When we defined exposure using an ITT approach, the crude incidence rate of all-cause, in-hospital mortality was 5.83 per 1,000 person-days (95% CI: 2.80, 10.72) among those receiving anticoagulants and 1.36 per 1,000 person-days (95% CI: 1.14, 1.59) among those who did not (Table 3; Supplementary Figure S2). Patients who received anticoagulants at cohort entry had a higher risk of mortality than those who did not (crude HR: 3.79, 95% CI: 1.99, 7.22) (Table 3). After adjusting for baseline covariates, the hazard ratio decreased but was accompanied by a wide 95% CI (HR: 1.12, 95% CI: 0.48, 2.64). The incidence rate (per 1,000 person-days) for the composite adverse event outcome was 3.20 (95% CI: 1.04, 7.47) among those who received anticoagulants at cohort entry and 1.80 (95% CI: 1.54, 2.08) among those who did not (Table 3; Supplementary Figure S2). The adjusted HR was 0.79 (95% CI: 0.28, 2.18). The distributions of the individual outcomes that were included in the composite outcome by the treatment groups are listed in Tables 3, 4; Supplementary Table S1. We also observed similar findings when exposure was defined using a time-varying approach (Table 4).

**TABLE 3:** Hazard ratios of adverse outcomes comparing use of anticoagulants versus non-use of anticoagulants among patients hospitalized with COVID-19 (Intention-to-treat exposure definition)^a^.

Exposure	No. of patients	No. of events	No. of person- days	Incidence rate^b^ (95% CI)	Hazard ratio (95% CI)	
					Crude	Adjusted^f^
All-cause in-hospital mortality						
Non-use of anticoagulants	2,653	146	107,702	1.36 (1.14–1.59)	(References)	(References)
Use of anticoagulants	24	10	1,715	5.83 (2.80–10.72)	3.79 (1.99–7.22)	1.12 (0.48–2.64)
Composite outcome^c^						
Non-use of anticoagulants	2,653	176	97,864	1.80 (1.54–2.08)	(References)	(References)
Use of anticoagulants	24	5	1,562	3.20 (1.04–7.47)	1.55 (0.64–3.78)	0.79 (0.28–2.18)
Respiratory outcomes^d^						
Non-use of anticoagulants	2,653	25	106,979	0.23 (0.15–0.34)	(References)	(References)
Use of anticoagulants	24	0	1,715	—	—	—
Cardiovascular outcomes^e^						
Non-use of anticoagulants	2,653	88	101,343	0.87 (0.70–1.07)	(References)	(References)
Use of anticoagulants	24	2	1,614	1.24 (0.15–4.48)	1.34 (0.33–5.46)	0.80 (0.17–3.76)
VTE						
Non-use of anticoagulants	2,653	1	107,620	0.01 (0.00–0.05)	(References)	(References)
Use of anticoagulants	24	0	1,715	—	—	—
Major bleeding						
Non-use of anticoagulants	2,653	43	104,439	0.41 (0.30–0.55)	(References)	(References)
Use of anticoagulants	24	0	1,680	—	—	—
ICU admission						
Non-use of anticoagulants	2,653	77	105,591	0.73 (0.58–0.91)	(References)	(References)
Use of anticoagulants	24	3	1,698	1.77 (0.36–5.16)	2.03 (0.64–6.45)	2.48 (0.60–10.33)

^a^ person-time was classified as either use of anticoagulants or non-use of anticoagulants based on if they received anticoagulants (oral or parenteral form) at the cohort entry.

^b^ Per 1,000 person-days.

^c^ Including respiratory outcome, cardiovascular outcome, VTE, major bleeding; ICU, admission.

^d^ Including acute respiratory distress syndrome, respiratory failure, ventilator use.

^e^ Including myocardial infarction, cardiac arrest, heart failure, stroke.

^f^ Adjusted for baseline covariates including age, sex, calendar time of cohort entry, type of insurance, numbers of unique medication use, numbers of hospitalization, comorbidities (obesity, hypertension, chronic kidney disease, diabetes, coronary artery disease, cerebrovascular disease, atrial fibrillation, heart failure, stroke, myocardial infarction, mechanical heart valves installation, coronary revascularization, venous thromboembolism, cancers, bleeding), medication use (antidiabetic drugs, antihypertensive drugs, nonsteroidal anti-inflammatory drugs, antiplatelets, statins, immunosuppressants, antivirals, corticosteroids, remdesivir), surgeries.

**TABLE 4:** Hazard ratios of outcomes comparing current use of anticoagulants versus non-current use of anticoagulants among patients hospitalized with COVID-19 (time-varying exposure definition)^a^.

Exposure	No. of patients	No. of events	No. of person- days	Incidence rate^b^ (95% CI)	Hazard ratio (95% CI)	
					Crude	Adjusted^f^
All-cause in-hospital mortality						
Non-current use of anticoagulants	2,662	149	107,948	1.38 (1.17–1.62)	(References)	(References)
Current use of anticoagulants	44	7	1,469	4.77 (1.92–9.82)	2.98 (1.39–6.38)	0.82 (0.33–2.07)
Composite outcome^c^						
Non-current use of anticoagulants	2,662	176	98,056	1.79 (1.54–2.08)	(References)	(References)
Current use of anticoagulants	44	5	1,370	3.65 (1.19–8.52)	1.61 (0.66–3.92)	0.60 (0.20–1.83)
Respiratory outcomes^d^						
Non-current use of anticoagulants	2,662	25	107,225	0.23 (0.15–0.34)	(References)	(References)
Current use of anticoagulants	44	0	1,469	—	—	—
Cardiovascular outcomes^e^						
Non-current use of anticoagulants	2,662	87	101,552	0.86 (0.69–1.06)	(References)	(References)
Current use of anticoagulants	44	3	1,405	2.14 (0.44–6.24)	2.15 (0.68–6.82)	0.54 (0.11–2.75)
VTE						
Non-current use of anticoagulants	2,662	1	107,866	0.01 (0.00–0.05)	(References)	(References)
Current use of anticoagulants	44	0	1,469	—	—	—
Major bleeding						
Non-current use of anticoagulants	2,662	43	104,671	0.41 (0.30–0.55)	(References)	(References)
Current use of anticoagulants	44	0	1,448	—	—	—
ICU admission						
Non-current use of anticoagulants	2,662	78	105,834	0.74 (0.58–0.92)	(References)	(References)
Current use of anticoagulants	44	2	1,455	1.37 (0.17–4.97)	1.49 (0.37–6.09)	1.89 (0.37–9.60)

^a^ Exposure status was updated daily with current use defined by the prescription for any anticoagulant on the day for which the exposure was being defined.

^b^ Per 1,000 person-days.

^c^ Including respiratory outcome, cardiovascular outcome, VTE, major bleeding; ICU, admission.

^d^ Including acute respiratory distress syndrome, respiratory failure, ventilator use.

^e^ Including myocardial infarction, cardiac arrest, heart failure, stroke.

^f^ Adjusted for baseline covariates including age, sex, calendar time of cohort entry, type of insurance, numbers of unique medication use, numbers of hospitalization, comorbidities (obesity, hypertension, chronic kidney disease, diabetes, coronary artery disease, cerebrovascular disease, atrial fibrillation, heart failure, stroke, myocardial infarction, mechanical heart valves installation, coronary revascularization, venous thromboembolism, cancers, bleeding), medication use (antidiabetic drugs, antihypertensive drugs, nonsteroidal anti-inflammatory drugs, antiplatelets, statins, immunosuppressants, antivirals, corticosteroids, remdesivir), surgeries.

## Discussion

In this nationwide cohort of patients hospitalized with COVID-19 in South Korea, a small proportion received anticoagulants during hospitalization. Substantial differences in demographic and clinical characteristics were present between treatment groups; patients who received anticoagulants were, on average, more than 20 years older and had worse baseline health compared to those who did not receive anticoagulants. Therefore, both use of anticoagulants at the cohort entry and (time-varying) current use of anticoagulants were associated with higher crude risks of mortality and adverse outcomes. The adjusted estimates were inconclusive, however, largely due to small numbers of events in the anticoagulant group.

Important differences exist between our study population and those of previous related studies. Our cohort was much younger (median age <50 years) than the study populations of studies conducted in the U.S. (Nadkarni et al., 2020), Spain (Ayerbe et al., 2020), China (Tang et al., 2020), and Italy (Russo et al., 2020) (median age >65 years). Studies have shown that older persons are at higher risk of hospitalization for COVID-19 (CDC Cases et al., 2020; Palmer et al., 2021), however the younger median age in our study may be a result of South Korean public health policies enacted in response to the COVID-19 outbreak. Early during the pandemic, all confirmed patients were hospitalized for isolation in South Korea (Chang et al., 2020; MOHW, 2021). Starting from March 2020, a four-stage patient classification system was applied based on disease severity, where moderate, severe, and extremely severe cases were required to be admitted to hospitals (Chang et al., 2020; MOHW, 2021). Therefore, our generally younger cohort may represent a heterogeneous population with different disease severities. The South Korean policy to also hospitalize individuals with relatively mild COVID-19, combined with younger age being associated with milder COVID-19 (Pijls et al., 2021), may explain why our study population is younger than the study populations of previous reported studies in this area. This may also account for the smaller number of patients receiving anticoagulants in our study, relative to previous COVID-19 studies examining anticoagulants.

If patients receiving anticoagulants were distinct from those not receiving anticoagulants in our study, the positivity assumption may have been violated. The positivity assumption, a key assumption for causal inference, holds when patients in each stratum of all covariate combinations have a chance of being in either treatment group (receiving or not receiving anticoagulants in the present study) (Petersen et al., 2012). This positivity violation is apparent from our comparison of the distributions of propensity score of receiving anticoagulants based on patient characteristics; the propensity score distributions did not overlap well between the two groups (Supplementary Figure S2A). When we restricted the population to the areas of propensity score overlap, approximately half of the patients were removed (Supplementary Figure S2B). The violation of the positivity assumption may have occurred because of the small number of anticoagulant users or because of a lack of clinical equipoise for using anticoagulants among patients with certain characteristics (Westreich and Cole, 2010; Platt et al., 2012). This latter reason may have been exaggerated by rapidly evolving COVID-19 treatment strategies based on emerging evidence or guidelines (Thachil et al., 2020). One study (Lin et al., 2020) using data from a large health care system in Massachusetts, U.S. showed that therapeutic choices for hospitalized patients with COVID-19 varied by disease severity and by calendar time (weekly). The positivity violation posed analytic challenges, especially when using inverse-probability weighted-based methods and problems with interpretation in general. Several alternative approaches can be applied (e.g., restriction, choosing a different estimand) (Petersen et al., 2012), however the small number of exposed patients limited the feasibility of these options. Although we applied regression in our study, cautious interpretation is warranted for making extrapolations in data-sparse regions (Vittinghoff and McCulloch, 2007).

Several observational studies have provided cumulative evidence on the association between anticoagulant use and COVID-19 outcomes, which has led to the initiation of several clinical trials (ATTACC Investigators, 2021; Inspiration Investigators, 2021; REMAP-CAP Investigators, 2021; Talasaz et al., 2021). A recent meta-analysis combining results from 19 observational studies showed that the use versus non-use of heparin improved all-cause mortality among hospitalized patients with COVID-19 (pooled HR: 0.66, 95% CI: 0.61, 0.72) (Giossi et al., 2021). However, with anticoagulant use varying over time during hospitalization, defining the exposure has posed challenges in the current literature. One of the most common design flaws in this literature is grouping any anticoagulant use during follow-up (Albani et al., 2020; Lynn et al., 2020; Paranjpe et al., 2020; Tang et al., 2020; Di Castelnuovo et al., 2021; Hara et al., 2021; Ionescu et al., 2021) rather than classifying exposure using a time-varying approach; this approach may introduce immortal time bias (Suissa, 2008). Currently, only a few studies with larger sample sizes have examined in-hospital prognosis of patients with COVID-19 with use versus non-use of anticoagulants. Nadkarni et al. (2020) found that compared to non-use of anticoagulants, use of prophylactic anticoagulants (HR: 0.53, 95% CI: 0.45, 0.62) or therapeutic anticoagulants (HR: 0.72, 95% CI: 0.58, 0.89) were associated with lower in-hospital mortality among 4,389 patients in a single U.S. hospital system. Although the authors acknowledged using time-varying exposure to avoid immortal-time bias, the time-varying exposure definition was not clearly reported. Another study (Rentsch et al., 2021) used data from a nationwide U.S. veterans’ health care system (*n* = 4,297); they found early initiation of prophylactic anticoagulants was associated with a decreased risk of 30-day mortality (HR: 0.73, 95% CI: 0.66, 0.81). Due to data limitations, the authors were not able to measure time-varying in-hospital use of anticoagulants. Similarly, a study conducted in a single U.S. medical center (*n* = 3,625) (Billett et al., 2020) adopted an ITT approach, defining anticoagulant use based on whether they received any within 48 h of admission. They found, that compared to those not receiving any anticoagulants, prophylactic apixaban use (OR: 0.46, 95% CI: 0.30, 0.71) and therapeutic apixaban (OR: 0.57, 95% CI: 0.38, 0.85) were associated with lower in-hospital mortality. However, this study excluded patients who remained hospitalized at the end of study period which may introduce selection bias.

The data source used for our study presented a unique opportunity for studying COVID-19 treatment repurposing since it was the first large nationwide COVID-19 database that included information on in-hospital medication use. However, there were some potential limitations. First, as a claims-based database, the diagnosis codes used to define outcomes were used for both clinical and billing purposes. Therefore, some outcome misclassification is possible, although the proportion of misclassified patients should be minimal since the outcomes were mostly severe in nature. Second, our results provide real-world evidence that reflects the situation of routine clinical practice in South Korea. These results may not be generalizable to other countries with different types of health care systems (Blumenthal et al., 2020) and different public health approaches to managing COVID-19. Third, we were unable to adjust for severity of COVID-19 upon hospital admission because laboratory data and scales of severity based on clinical consensus were not available. However, we approximated the underlying health condition for the patients based on comprehensive comorbidities and medication use history. Fourth, there was potential confounding by time-dependent variables in our study. Although we adopted time-varying approaches to define treatment status and the covariates, we did not include time-varying covariates in our final analyses due to data limitations (small number of patients using anticoagulants). If patients with arising comorbidities or with more severe symptoms during hospitalization were more likely to be prescribed anticoagulants, the benefits of anticoagulants would be underestimated in our study. However, in practice, the direction of bias may be difficult to articulate due to the complex scenarios. For example, some of these time-varying covariates were likely affected by the past use of anticoagulants and were also the confounders for subsequent anticoagulant use and the outcome. In a future study, it is important to apply the analytic approaches that could appropriately account for this type of time-varying confounders, such as g-methods (Mansournia et al., 2017) (including a marginal structure model which was listed as our original planned analyses in Supplementary Appendix SA3) instead of a traditional time-varying Cox proportional hazard model (Hernán et al., 2000). In addition, the hazard ratios from the Cox proportional hazard model may not have a causal interpretation since they were estimated conditional on survival which was different from the parameters estimated from the marginal structural model (Hernán, 2010; Martinussen et al., 2020). Fifth, it was not feasible to define anticoagulant use as prophylactic or therapeutic, since we lacked information on clinical protocols and notes. Sixth, our study did not exclude patients who used anticoagulants within 1 year prior to cohort entry (known as prevalent users), which may potentially introduce selection bias (Ray, 2003). Patients are usually at a higher risk of bleeding within the first few months of initiation of anticoagulants (Khan et al., 2019). Therefore, prevalent users of anticoagulants may have a depletion of susceptibility of major bleeding (one of the secondary outcomes), potentially resulting in a lower risk of major bleeding compared to those who newly initiated anticoagulants. However, it is unknown whether there is a depletion of susceptibility of adverse health outcomes in our study among prevalent users of anticoagulants. If these biases were present, our study results would overestimate the benefits of the anticoagulants on COVID-19 related outcomes.

Our study demonstrated that the rapid evolution of the COVID-19 pandemic may have important implications for the study of COVID-19 treatment effectiveness using existing data infrastructure. The small portion of patients who received anticoagulants in our study may result from the combinations of the quarantine policies and clinical management during the early stage of COVID-19 pandemic in South Korea. The study period was during the early stages of the COVID-19 pandemic in one of the first areas affected worldwide. Since then, clinical care for COVID-19 has changed rapidly during this dynamic pandemic as new research findings emerged, vaccines were developed and widely adopted starting in 2021, and we developed a better understanding of the disease course. It is essential to leverage data sources (e.g., electronic medical records) that can capture relevant time-varying information, such as detailed clinical measurements, laboratory test results and clinical procedures, to address confounding as clinical and public health practice evolved during the pandemic (Pottegård et al., 2020). In addition, jurisdiction-specific policies, medical system capacity, and vaccination rates may impact the heterogeneity in hospitalized patients’ disease severity in different regions, all of which evolved over time. All of these factors need to be considered when interpreting and comparing results. Several initiatives have been launched to provide timely access to the large databases with multiple data sources, such as the International 4CE Consortium (Brat et al., 2020), National COVID Cohort Collaborative (N3C) (Bennett et al., 2021) in the U.S., and the database used in our study. With the appropriate data sources in place, investigators from multiple disciplines, and rigorous study design (Franklin et al., 2021; Powell et al., 2021), observational studies can be useful to help inform the constantly evolving evidence on treatments for COVID-19.

## Conclusion

Although our study was unable to draw conclusions on the effectiveness of anticoagulants on COVID-19 prognosis due to the small number of patients who received anticoagulants during hospitalization, these results can contribute to future knowledge syntheses for this important question. Our study demonstrated that the dynamic pandemic environment may have important implications for observational studies of COVID-19 treatment effectiveness. These results highlighted the need for investigators of future studies to be aware of methodological challenges and to leverage partnerships to access appropriate data sources.

## Data Availability

The datasets presented in this article are not readily available because the data that support the findings of this study are available from the Health Insurance Review and Assessment Service of South Korea. These data are not publicly available because of restrictions that apply to the availability of these data based on domestic laws and regulations that prohibit the distribution or release of an individual’s data to the public. Requests to access the datasets should be directed to the Health Insurance Review and Assessment Service of South Korea (globalmaster@hiramail.net).

## References

[B1] Albani F. Sepe L. Fusina F. Prezioso C. Baronio M. Caminiti F. (2020). Thromboprophylaxis with enoxaparin is associated with a lower death rate in patients hospitalized with SARS-CoV-2 infection. A cohort study. EClinicalMedicine 27, 100562. 10.1016/j.eclinm.2020.100562 33043287 PMC7534836

[B2] ATTACC Investigators (2021). Therapeutic anticoagulation with heparin in noncritically ill patients with covid-19. N. Engl. J. Med. 385, 790–802. 10.1056/nejmoa2105911 34351721 PMC8362594

[B3] Austin P. C. (2009). Balance diagnostics for comparing the distribution of baseline covariates between treatment groups in propensity-score matched samples. Stat. Med. 28, 3083–3107. 10.1002/sim.3697 19757444 PMC3472075

[B4] Ayerbe L. Risco C. Ayis S. (2020). The association between treatment with heparin and survival in patients with Covid-19. J. Thromb. Thrombolysis 50, 298–301. 10.1007/s11239-020-02162-z 32476080 PMC7261349

[B5] Bennett T. D. Moffitt R. A. Hajagos J. G. Amor B. Anand A. Bissell M. M. (2021). Clinical characterization and prediction of clinical severity of SARS-CoV-2 infection among US adults using data from the US national COVID cohort collaborative. JAMA Netw. Open 4, e2116901. 10.1001/jamanetworkopen.2021.16901 34255046 PMC8278272

[B6] Billett H. H. Reyes-Gil M. Szymanski J. Ikemura K. Stahl L. R. Lo Y. (2020). Anticoagulation in COVID-19: Effect of enoxaparin, heparin, and apixaban on mortality. Thromb. Haemost. 120, 1691–1699. 10.1055/s-0040-1720978 33186991 PMC7869055

[B7] Blumenthal D. Fowler E. J. Abrams M. Collins S. R. (2020). Covid-19 — implications for the health care system. N. Engl. J. Med. 383, 1483–1488. 10.1056/NEJMsb2021088 32706956

[B8] Brat G. A. Weber G. M. Gehlenborg N. Avillach P. Palmer N. P. Chiovato L. (2020). International electronic health record-derived COVID-19 clinical course profiles: the 4CE consortium. Npj Digit. Med. 3, 109–9. 10.1038/s41746-020-00308-0 32864472 PMC7438496

[B9] CDC Cases, Data, and Surveillance (2020). Centers for disease control and prevention Available at: https://www.cdc.gov/coronavirus/2019-ncov/covid-data/investigations-discovery/hospitalization-death-by-age.html .

[B10] Chang M. C. Baek J. H. Park D. (2020). Lessons from South Korea regarding the early stage of the COVID-19 outbreak. Healthcare 8, E229. 10.3390/healthcare8030229 PMC7551130 32722174

[B11] Cohen J. B. D’Agostino McGowan L. Jensen E. T. Rigdon J. South A. M. (2021). Evaluating sources of bias in observational studies of angiotensin-converting enzyme inhibitor/angiotensin II receptor blocker use during COVID-19: beyond confounding. J. Hypertens. 39, 795–805. 10.1097/HJH.0000000000002706 33186321 PMC8164085

[B12] Di Castelnuovo A. Costanzo S. Antinori A. Berselli N. Blandi L. Bonaccio M. (2021). Heparin in COVID-19 patients is associated with reduced in-hospital mortality: the multicenter Italian CORIST study. Thromb. Haemost. 121, 1054–1065. 10.1055/a-1347-6070 33412596

[B13] Franklin J. M. Gatto N. M. Rassen J. A. Glynn R. J. Schneeweiss S. (2021). Real-world evidence for assessing pharmaceutical treatments in the context of COVID-19. Clin. Pharmacol. Ther. 109, 816–828. 10.1002/cpt.2185 33529354 PMC8014840

[B14] Giossi R. Menichelli D. Pani A. Tratta E. Romandini A. Roncato R. (2021). A systematic review and a meta-analysis comparing prophylactic and therapeutic low molecular weight heparins for mortality reduction in 32, 688 COVID-19 patients. Front. Pharmacol. 0. 10.3389/fphar.2021.698008 PMC8443784 34539396

[B15] Griffith G. J. Morris T. T. Tudball M. J. Herbert A. Mancano G. Pike L. (2020). Collider bias undermines our understanding of COVID-19 disease risk and severity. Nat. Commun. 11, 5749. 10.1038/s41467-020-19478-2 33184277 PMC7665028

[B16] Hara H. Uemura Y. Hayakawa K. Togano T. Asai Y. Matsunaga N. (2021). Evaluation of the efficacy of anticoagulation therapy in reducing mortality in a nationwide cohort of hospitalized patients with coronavirus disease in Japan. Int. J. Infect. Dis. 112, 111–116. 10.1016/j.ijid.2021.09.014 34517044 PMC8432973

[B17] Hernán M. A. Robins J. M. (2016). Using big data to emulate a target trial when a randomized trial is not available. Am. J. Epidemiol. 183, 758–764. 10.1093/aje/kwv254 26994063 PMC4832051

[B18] Hernán M. Á. Brumback B. Robins J. M. (2000). Marginal structural models to estimate the causal effect of zidovudine on the survival of HIV-positive men. Epidemiology 11, 561–570. 10.1097/00001648-200009000-00012 10955409

[B19] Hernán M. A. (2010). The hazards of hazard ratios. Epidemiology 21, 13–15. 10.1097/EDE.0b013e3181c1ea43 20010207 PMC3653612

[B20] Hoffman K. L. Schenck E. J. Satlin M. J. Whalen W. Pan D. Williams N. (2022). Comparison of a target trial emulation framework vs Cox regression to estimate the association of corticosteroids with COVID-19 mortality. JAMA Netw. Open 5, e2234425. 10.1001/jamanetworkopen.2022.34425 36190729 PMC9530966

[B21] Iba T. Levy J. H. Levi M. Thachil J. (2020). Coagulopathy in COVID-19. J. Thromb. Haemost. 18, 2103–2109. 10.1111/jth.14975 32558075 PMC7323352

[B22] Inspiration Investigators (2021). Effect of intermediate-dose vs standard-dose prophylactic anticoagulation on thrombotic events, extracorporeal membrane oxygenation treatment, or mortality among patients with COVID-19 admitted to the intensive care unit: The INSPIRATION randomized clinical trial. JAMA 325, 1620–1630. 10.1001/jama.2021.4152 33734299 PMC7974835

[B23] Ionescu F. Jaiyesimi I. Petrescu I. Lawler P. R. Castillo E. Munoz-Maldonado Y. (2021). Association of anticoagulation dose and survival in hospitalized COVID-19 patients: a retrospective propensity score-weighted analysis. Eur. J. Haematol. 106, 165–174. 10.1111/ejh.13533 33043484 PMC7675265

[B24] Jiménez D. Garcia-Sanchez A. Rali P. Muriel A. Bikdeli B. Ruiz-Artacho P. (2021). Incidence of VTE and bleeding among hospitalized patients with coronavirus disease 2019: a systematic review and meta-analysis. Chest 159, 1182–1196. 10.1016/j.chest.2020.11.005 33217420 PMC7670889

[B25] Khan F. Kimpton M. Tritschler T. Le Gal G. Hutton B. Fergusson D. A. (2019). Risk of major bleeding during extended oral anticoagulation in patients with first unprovoked venous thromboembolism: a systematic review and meta-analysis protocol. Syst. Rev. 8, 245. 10.1186/s13643-019-1175-5 31661033 PMC6819358

[B26] Khani E. Khiali S. Entezari‐Maleki T. (2021). Potential COVID‐19 therapeutic agents and vaccines: an evidence‐based review. J. Clin. Pharmacol. 61, 429–460. 10.1002/jcph.1822 33511638 PMC8014753

[B27] Kim J. A. Yoon S. Kim L. Y. Kim D. S. (2017). Towards actualizing the value potential of Korea health insurance review and assessment (HIRA) data as a resource for health research: Strengths, limitations, applications, and strategies for optimal use of HIRA data. J. Korean Med. Sci. 32, 718–728. 10.3346/jkms.2017.32.5.718 28378543 PMC5383602

[B28] Kyoung D.-S. Kim H.-S. (2022). Understanding and utilizing claim data from the Korean national health insurance service (NHIS) and health insurance review & assessment (HIRA) database for research. J. Lipid Atheroscler. 11, 103–110. 10.12997/jla.2022.11.2.103 35656154 PMC9133780

[B29] Lin K. J. Schneeweiss S. Tesfaye H. D'Andrea E. Liu J. Lii J. (2020). Pharmacotherapy for hospitalized patients with COVID-19: Treatment patterns by disease severity. Drugs 80, 1961–1972. 10.1007/s40265-020-01424-7 33151482 PMC7643089

[B30] Lippi G. Sanchis-Gomar F. Favaloro E. J. Lavie C. J. Henry B. M. (2021). Coronavirus disease 2019–associated coagulopathy. Mayo Clin. Proc. 96, 203–217. 10.1016/j.mayocp.2020.10.031 33413819 PMC7604017

[B31] Lynn L. Reyes J. A. Hawkins K. Panda A. Linville L. Aldhahri W. (2020). The effect of anticoagulation on clinical outcomes in novel Coronavirus (COVID-19) pneumonia in a U.S. cohort. Thromb. Res. 197, 65–68. 10.1016/j.thromres.2020.10.031 33186849 PMC7644262

[B32] Mansournia M. A. Etminan M. Danaei G. Kaufman J. S. Collins G. (2017). Handling time varying confounding in observational research. BMJ 359, j4587. 10.1136/bmj.j4587 29038130

[B33] Martinussen T. Vansteelandt S. Andersen P. K. (2020). Subtleties in the interpretation of hazard contrasts. Lifetime Data Anal. 26, 833–855. 10.1007/s10985-020-09501-5 32654089

[B34] MOHW (2021). Coronavirus disease 19(COVID-19). Available at: http://ncov.mohw.go.kr/en/ .

[B35] Nadkarni G. N. Lala A. Bagiella E. Chang H. L. Moreno P. R. Pujadas E. (2020). Anticoagulation, bleeding, mortality, and pathology in hospitalized patients with COVID-19. J. Am. Coll. Cardiol. 76, 1815–1826. 10.1016/j.jacc.2020.08.041 32860872 PMC7449655

[B36] Nemeth B. Lijfering W. M. Nelissen R. G. H. H. Schipper I. B. Rosendaal F. R. le Cessie S. (2019). Risk and risk factors associated with recurrent venous thromboembolism following surgery in patients with history of venous thromboembolism. JAMA Netw. Open 2, e193690. 10.1001/jamanetworkopen.2019.3690 31074822 PMC6512304

[B37] Palmer S. Cunniffe N. Donnelly R. (2021). COVID-19 hospitalization rates rise exponentially with age, inversely proportional to thymic T-cell production. J. R. Soc. Interface 18, 20200982. 10.1098/rsif.2020.0982 33726544 PMC8086881

[B38] Paranjpe I. Fuster V. Lala A. Russak A. J. Glicksberg B. S. Levin M. A. (2020). Association of treatment dose anticoagulation with in-hospital survival among hospitalized patients with COVID-19. J. Am. Coll. Cardiol. 76, 122–124. 10.1016/j.jacc.2020.05.001 32387623 PMC7202841

[B39] Petersen M. L. Porter K. E. Gruber S. Wang Y. van der Laan M. J. (2012). Diagnosing and responding to violations in the positivity assumption. Stat. Methods Med. Res. 21, 31–54. 10.1177/0962280210386207 21030422 PMC4107929

[B40] Pijls B. G. Jolani S. Atherley A. Derckx R. T. Dijkstra J. I. R. Franssen G. H. L. (2021). Demographic risk factors for COVID-19 infection, severity, ICU admission and death: a meta-analysis of 59 studies. BMJ Open 11, e044640. 10.1136/bmjopen-2020-044640 PMC7802392 33431495

[B41] Platt R. W. Delaney J. A. C. Suissa S. (2012). The positivity assumption and marginal structural models: the example of warfarin use and risk of bleeding. Eur. J. Epidemiol. 27, 77–83. 10.1007/s10654-011-9637-7 22160333

[B42] Pottegård A. Kurz X. Moore N. Christiansen C. F. Klungel O. (2020). Considerations for pharmacoepidemiological analyses in the SARS-CoV-2 pandemic. Pharmacoepidemiol. Drug Saf. 29, 825–831. 10.1002/pds.5029 32369865

[B43] Powell M. Koenecke A. Byrd J. B. Nishimura A. Konig M. F. Xiong R. (2021). Ten rules for conducting retrospective pharmacoepidemiological analyses: Example COVID-19 study. Front. Pharmacol. 12, 700776. 10.3389/fphar.2021.700776 34393782 PMC8357144

[B44] Ray W. A. (2003). Evaluating medication effects outside of clinical trials: new-user designs. Am. J. Epidemiol. 158, 915–920. 10.1093/aje/kwg231 14585769

[B45] REMAP-CAP Investigators (2021). Therapeutic anticoagulation with heparin in critically ill patients with covid-19. N. Engl. J. Med. 385, 777–789. 10.1056/nejmoa2103417 34351722 PMC8362592

[B46] Renoux C. Azoulay L. Suissa S. (2021). Biases in evaluating the safety and effectiveness of drugs for the treatment of COVID-19: designing real-world evidence studies. Am. J. Epidemiol. 190, 1452–1456. 10.1093/aje/kwab028 33564823 PMC7929453

[B47] Rentsch C. T. Beckman J. A. Tomlinson L. Gellad W. F. Alcorn C. Kidwai-Khan F. (2021). Early initiation of prophylactic anticoagulation for prevention of coronavirus disease 2019 mortality in patients admitted to hospital in the United States: cohort study. BMJ 372, n311. 10.1136/bmj.n311 33574135 PMC7876672

[B48] Rho Y. Cho D. Y. Son Y. Lee Y. J. Kim J. W. Lee H. J. (2021). COVID-19 international collaborative research by the health insurance review and assessment service using its nationwide real-world data: Database, outcomes, and implications. J. Prev. Med. Public Health Yebang Uihakhoe Chi 54, 8–16. 10.3961/jpmph.20.616 33618494 PMC7939755

[B49] Russo V. Di Maio M. Attena E. Silverio A. Scudiero F. Celentani D. (2020). Clinical impact of pre-admission antithrombotic therapy in hospitalized patients with COVID-19: A multicenter observational study. Pharmacol. Res. 159, 104965. 10.1016/j.phrs.2020.104965 32474087 PMC7256617

[B50] Suissa S. (2008). Immortal time bias in pharmaco-epidemiology. Am. J. Epidemiol. 167, 492–499. 10.1093/aje/kwm324 18056625

[B51] Talasaz A. H. Sadeghipour P. Kakavand H. Aghakouchakzadeh M. Kordzadeh-Kermani E. Van Tassell B. W. (2021). Recent randomized trials of antithrombotic therapy for patients with COVID-19: JACC state-of-the-art review. J. Am. Coll. Cardiol. 77, 1903–1921. 10.1016/j.jacc.2021.02.035 33741176 PMC7963001

[B52] Tang N. Bai H. Chen X. Gong J. Li D. Sun Z. (2020). Anticoagulant treatment is associated with decreased mortality in severe coronavirus disease 2019 patients with coagulopathy. J. Thromb. Haemost. 18, 1094–1099. 10.1111/jth.14817 32220112 PMC9906401

[B53] Thachil J. Tang N. Gando S. Falanga A. Cattaneo M. Levi M. (2020). ISTH interim guidance on recognition and management of coagulopathy in COVID-19. J. Thromb. Haemost. 18, 1023–1026. 10.1111/jth.14810 32338827 PMC9906133

[B54] Vittinghoff E. McCulloch C. E. (2007). Relaxing the rule of ten events per variable in logistic and Cox regression. Am. J. Epidemiol. 165, 710–718. 10.1093/aje/kwk052 17182981

[B55] Westreich D. Cole S. R. (2010). Invited commentary: positivity in practice. Am. J. Epidemiol. 171, 674–677. 10.1093/aje/kwp436 20139125 PMC2877454

[B56] WHO (2020). Laboratory testing for coronavirus disease 2019 (COVID-19) in suspected human cases: interim guidance.Available at: https://apps.who.int/iris/handle/10665/331501 .

[B57] WHO (2022). WHO coronavirus (COVID-19) dashboard. Available at: https://covid19.who.int .

