# Supplementary material for: Challenges in evaluating treatments for COVID-19: The case of in-hospital anticoagulant use and the risk of adverse outcomes
Source: Front Pharmacol. 2022 Nov 24;13:1034636. doi: 10.3389/fphar.2022.1034636 (PMC9729259; doi:10.3389/fphar.2022.1034636)
Supplement: Supplementary file 2 [file Table3.DOCX]

**Appendix.**

Appendix 1. Hypothetical target trial

**Eligibility criteria**: Patients hospitalized with COVID-19, diagnosed based on the positive results from a reverse transcription polymerase chain reaction test. We would exclude patients age less than 20 years and those with active cancer at the time of hospitalization admission.

**Treatment intervention strategies and assignment**: At the time of admission to the hospital, patients were randomly assigned into either one of the two groups: use of anticoagulants or no use of anticoagulants. Anticoagulants included vitamin K antagonists, direct oral anticoagulants, heparin (low molecular weight heparin [LMWH] or unfractionated heparin [UFH]), and fondaparinux. Treatment duration would be from admission day throughout the entire hospitalization.

**Outcome**: (1) Primary outcome: In-hospital death (2) Composite secondary outcomes:

Respiratory outcomes (acute respiratory distress syndrome, respiratory failure, ventilator use), cardiovascular outcomes (myocardial infarction, cardiac arrest, heart failure, stroke), VTE (deep vein thrombosis, pulmonary embolism), major bleeding (intracranial hemorrhage, hemorrhagic stroke, gastrointestinal bleeding, other major bleeding), and ICU admission.

**Follow-up**: All the patients would be followed from the day of admission until an event or censoring due to hospital discharge, in-hospital death (for outcomes other than mortality), or the end of the study period, whichever occurred first.

**Causal contrasts**: Intentional-to-treat effect and per-protocol effect

Appendix 2. Variable lists

Adverse outcomes included in the composite secondary outcomes:

Respiratory outcomes (acute respiratory distress syndrome, respiratory failure, ventilator use), cardiovascular outcomes (myocardial infarction, cardiac arrest, heart failure, stroke), VTE (deep vein thrombosis, pulmonary embolism), major bleeding (intracranial hemorrhage, hemorrhagic stroke, gastrointestinal bleeding, other major bleeding), and ICU admission.

Potential confounders

Comorbidities: obesity, hypertension, chronic kidney disease, diabetes, coronary artery disease, cerebrovascular disease, atrial fibrillation, heart failure, stroke, myocardial infarction, mechanical heart valves installation, coronary revascularization, VTE, cancer [>1 year before cohort entry; other than non-melanoma skin cancer], bleeding.

Medication use: antidiabetic drugs, antihypertensive drugs, nonsteroidal anti-inflammatory drugs, antiplatelets, statins, immunosuppressants, antivirals, corticosteroids, remdesivir, hormone replacement therapy, testosterone, tamoxifen, selective serotonin reuptake inhibitor.

Appendix 3. Prior statistical analysis plan for the secondary and sensitivity analyses

Due to the small number of exposed patients, planned secondary and sensitivity analyses were deemed not feasible. However, to ensure transparently, the priori statistical plan for these analyses is reported below:

Secondary analysis

Marginal structural Cox models will be used to estimate hazard ratios and 95% confidence intervals for each outcome for current (instantaneous) use of anticoagulants versus non-use, adjusting for time-fixed and time-varying confounders. In brief, the statuses for treatment and covariates of our study will be measured daily. We will then use pooled logistic regression to pool persons and times to estimate the probability of receiving anticoagulants while conditioning on the baseline covariate, time-varying covariates, the treatment history of previous time point, and time as the denominator of the treatment weight. Continuous variables (e.g., age, time) will be modeled flexibly with restricted quadratic splines. These treatment weights will be stabilized by multiplying the probability of receiving anticoagulants, while conditioning on all covariates in the denominator model except for those time-varying covariates. The treatment weights will be multiplied cumulatively across time by patient. To account for informative censoring due to death (for non-fatal outcomes) or discharge from hospital, we will also estimate two censoring weights. Similar to the procedure mentioned above, instead of modeling the probability of receiving anticoagulants, we applied the same set of covariates to model the probability of censoring because of 1) in-hospital death; and 2) hospital discharge. Finally, we will adopt pooled logistic regression weighted by the multiplication product of treatment weight and censoring weight to approximate the corresponding hazard ratio: the outcome of interest comparing use of anticoagulants versus non-use estimated from the Marginal structural Cox model. The weighted Kaplan-Meier curves showing the cumulative incidence proportion of each outcome by treatment group will be plotted over the follow-up duration. We will also summarize the distributions of the treatment weights and censoring weights by follow-up time to determine if there are extreme weights and to detect positivity violations.

We will explore the outcomes of interest by type of anticoagulants (no current use of anticoagulants, current use of oral anticoagulants, current use of parenteral anticoagulants, current use of both oral and parenteral anticoagulants) and anticoagulant dosing (prophylactic, therapeutic dosage). We will also explore the duration-response relationship between cumulative anticoagulants use and the outcomes of interest by modeling the cumulative anticoagulants use as a flexible continuous term (e.g., restricted quadratic spline) in the outcome model. We will also repeat our primary analysis for each individual outcome within the composite outcomes, sample size permitting. The analyses will be considered as exploratory due to the limited number of included events. To investigate possible effect modification from medical history related to contraindication to anticoagulants, we will stratify our primary analysis by previous history of hospitalization for major bleeding (in the past 3 years) or heparin-induced thrombocytopenia during the year prior to cohort entry.

Sensitivity analysis

A series of sensitivity analyses will be conducted to confirm the robustness of our results. First, to mitigate the residual confounding from the lagged effects of the time-varying variables, we will repeat our analysis by including time-varying covariates that are lagged 5 days before being included in the models used to estimate treatment and censoring weights. Second, we will repeat the main analysis stratified by new-user versus prevalent user, which is defined as those who used anticoagulants during the year prior to cohort entry. Third, we will repeat our primary analysis with different definitions of current exposure. The alternative definitions will include defining current use as having the prescription for any anticoagulant during 1, 3 or 5 days prior to the day for which the exposure is being defined. Fourth, we will exclude patients with any history of cancer in the 3 years before cohort entry. Fifth, we will repeat our main analysis stratified by patients with major indications for anticoagulants at baseline, including atrial fibrillation, VTE, and recent major surgery versus without major indications.

**Table S1.** Distribution of type of anticoagulants use.

| **Exposure** | **No. of patients at cohort entry** | **No. of patients during follow-up** | **Person-days**  **(Time-varying approach)** |
| --- | --- | --- | --- |
| Use vs. non-use |  |  |  |
| Non-use of anticoagulants | 2,653 | 2,662 | 107,948 |
| Use of anticoagulants | 24 | 44 | 1,469 |
| Type of anticoagulants |  |  |  |
| Vitamin K antagonists | 6 | 6 | 485 |
| Direct oral anticoagulants | 11 | 19 | 746 |
| Low molecular weight heparin | 5 | 12 | 239 |
| Unfractionated heparin | 5 | 12 | 32 |
| Fondaparinux | 0 | 0 | 0 |
| Commination |  |  |  |
| Use of oral anticoagulants only | 13 | 16 | 1,193 |
| Use of parenteral anticoagulants only | 8 | 23 | 250 |
| Use of both oral and parenteral anticoagulants | 3 | 5 | 26 |

**Table S2.** Incidence rates of adverse outcomes by anticoagulants use among patients hospitalized with COVID-19 in South Korea.

|  | **Intention-to-treat approach**^a^ | | | | **Time-varying exposure approach**^b^ | | | |
| --- | --- | --- | --- | --- | --- | --- | --- | --- |
| **Outcomes** | **No. of patients** | **No. of events** | **No. of**  **Person-days** | **Incidence rate (95% CI)**^c^ | **No. of patients** | **No. of events** | **No. of**  **Person-days** | **Incidence rate (95% CI)**^c^ |
| *Respiratory outcomes* | | | | | | | | |
| **Acute respiratory distress syndrome** | | | | | | | | |
| Non-use of anticoagulants | 2,653 | 0 | 107,702 | - | 2,662 | 0 | 107,948 | - |
| Use of anticoagulants | 24 | 0 | 1,715 | - | 44 | 0 | 1,469 | - |
| **Respiratory failure** | | | | | | | | |
| Non-use of anticoagulants | 2,653 | 3 | 107,498 | 0.03 (0.01-0.08) | 2,662 | 3 | 107,744 | 0.03 (0.01-0.08) |
| Use of anticoagulants | 24 | 0 | 1,715 | - | 44 | 0 | 1,469 | - |
| **Ventilator use** | | | | | | | | |
| Non-use of anticoagulants | 2,653 | 22 | 107,183 | 0.21 (0.13-0.31) | 2,662 | 22 | 107,429 | 0.20 (0.13-0.31) |
| Use of anticoagulants | 24 | 0 | 1,715 | - | 44 | 0 | 1,469 | - |
| *Cardiovascular outcomes* | | | | | | | | |
| **Myocardial infarction** | | | | | | | | |
| Non-use of anticoagulants | 2,653 | 5 | 107,410 | 0.05 (0.02-0.11) | 2,662 | 5 | 107,656 | 0.05 (0.02-0.11) |
| Use of anticoagulants | 24 | 0 | 1,715 | - | 44 | 0 | 1,469 | - |
| **Cardiac arrest** | | | | | | | | |
| Non-use of anticoagulants | 2,653 | 3 | 107,679 | 0.03 (0.01-0.08) | 2,662 | 3 | 107,925 | 0.03 (0.01-0.08) |
| Use of anticoagulants | 24 | 0 | 1,715 | - | 44 | 0 | 1,469 | - |
| **Heart failure** | | | | | | | | |
| Non-use of anticoagulants | 2,653 | 12 | 107,130 | 0.11 (0.06-0.20) | 2,662 | 11 | 107,339 | 0.10 (0.05-0.18) |
| Use of anticoagulants | 24 | 1 | 1,652 | 0.61 (0.02-3.37) | 44 | 2 | 1,443 | 1.39 (0.17-5.01) |
| **Stroke** | | | | | | | | |
| Non-use of anticoagulants | 2,653 | 69 | 102,168 | 0.68 (0.53-0.85) | 2,662 | 69 | 102,414 | 0.67 (0.52-0.85) |
| Use of anticoagulants | 24 | 1 | 1,677 | 0.60 (0.02-3.32) | 44 | 1 | 1,431 | 0.70 (0.02-3.89) |

^a^ Person-time classified in to either use of anticoagulants or non-use of anticoagulants based on if they received anticoagulants (oral or parenteral form) at the cohort entry

^b^ Exposure status was updated daily with current use defined by the prescription for any anticoagulant on the day for which the exposure was being defined.

^b^ Per 1000 person-days

**Figure S1.** Study design and time windows for assessing variables

(The figure is adapted from Schneeweiss S, et al, Graphical Depiction of Longitudinal Study Designs in Health Care Databases. Ann Intern Med. 2019 Mar 19;170(6):398-406)

*
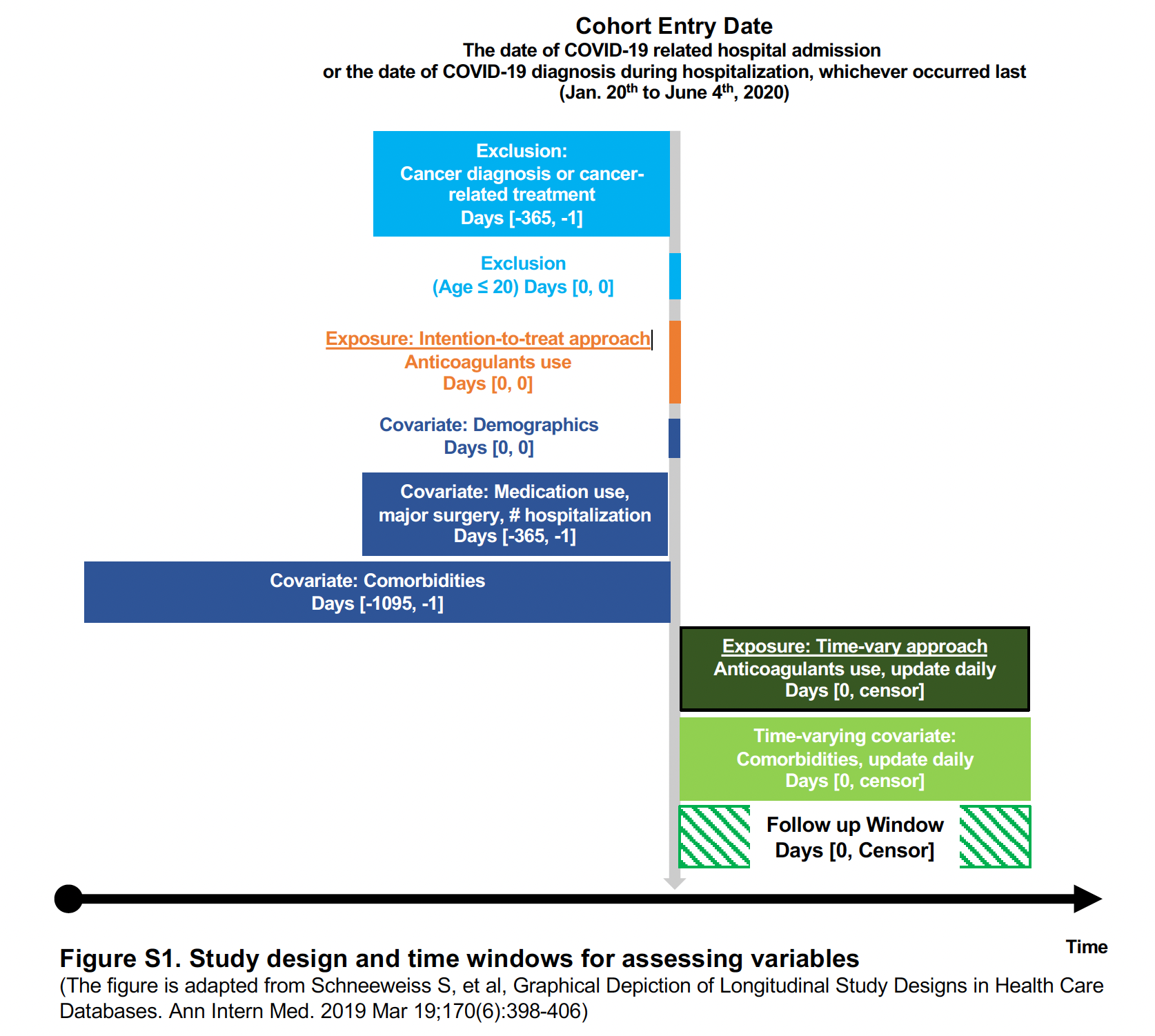
*

**Figure S2.** Kaplan-Meier curves for crude cumulative incidence proportion of each outcome by use of anticoagulants at the cohort entry.

| 1. All-cause in-hospital mortality |
| --- |
| 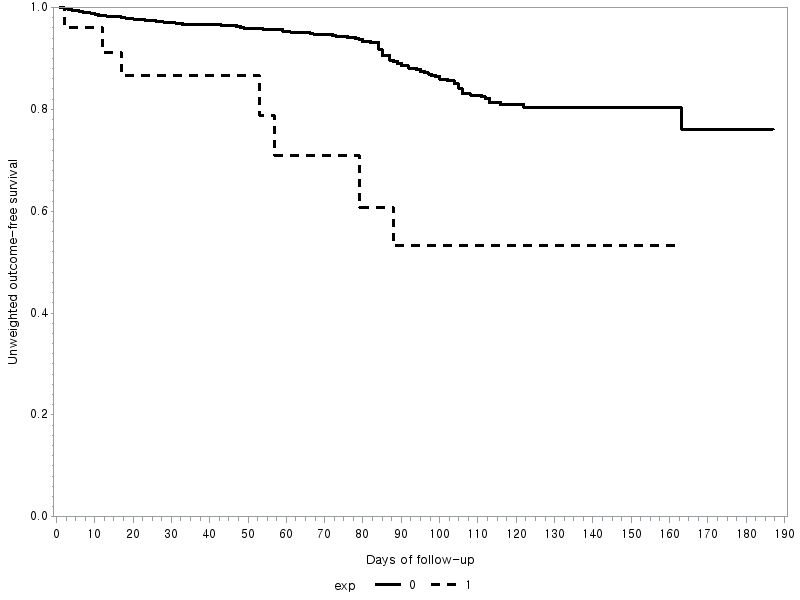   \| Number at risk \| Duration of follow-up (days) \| \| \| \| \| \| \| \| --- \| --- \| --- \| --- \| --- \| --- \| --- \| --- \| \|  \| 0 \| 30 \| 60 \| 90 \| 120 \| 150 \| 180 \| \| Non-exposed \| 2653 \| 1088 \| 551 \| 398 \| 175 \| 39 \| 1 \| \| Exposed \| 24 \| 15 \| 7 \| 6 \| 1 \| 1 \| 0 \| |
| 1. Composite outcome |
| 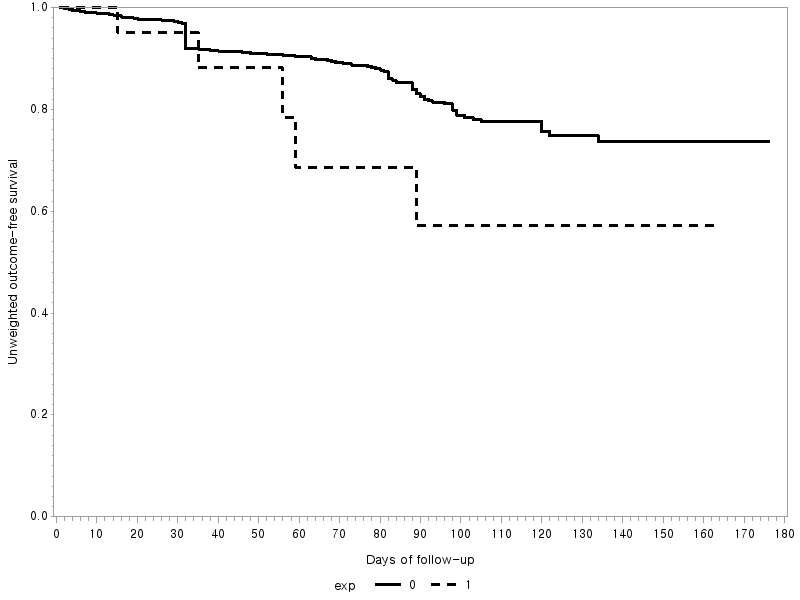   \| Number at risk \| Duration of follow-up (days) \| \| \| \| \| \| \| \| --- \| --- \| --- \| --- \| --- \| --- \| --- \| --- \| \|  \| 0 \| 30 \| 60 \| 90 \| 120 \| 150 \| 180 \| \| Non-exposed \| 2653 \| 1047 \| 463 \| 304 \| 117 \| 25 \| 0 \| \| Exposed \| 24 \| 14 \| 6 \| 4 \| 1 \| 1 \| 0 \| |
|  |
| 1. Respiratory outcomes |
| 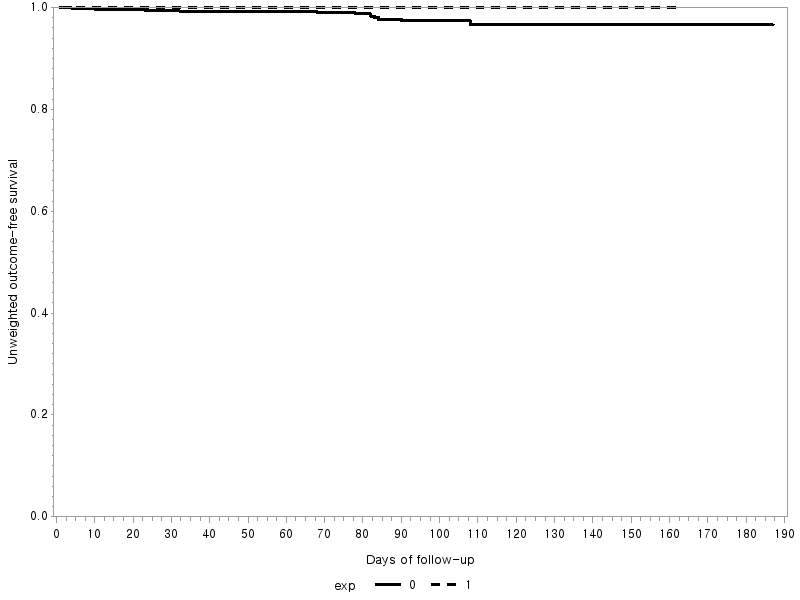   \| Number at risk \| Duration of follow-up (days) \| \| \| \| \| \| \| \| --- \| --- \| --- \| --- \| --- \| --- \| --- \| --- \| \|  \| 0 \| 30 \| 60 \| 90 \| 120 \| 150 \| 180 \| \| Non-exposed \| 2653 \| 1082 \| 545 \| 393 \| 171 \| 36 \| 1 \| \| Exposed \| 24 \| 15 \| 7 \| 6 \| 1 \| 1 \| 0 \| |
| 1. Cardiovascular outcomes |
| 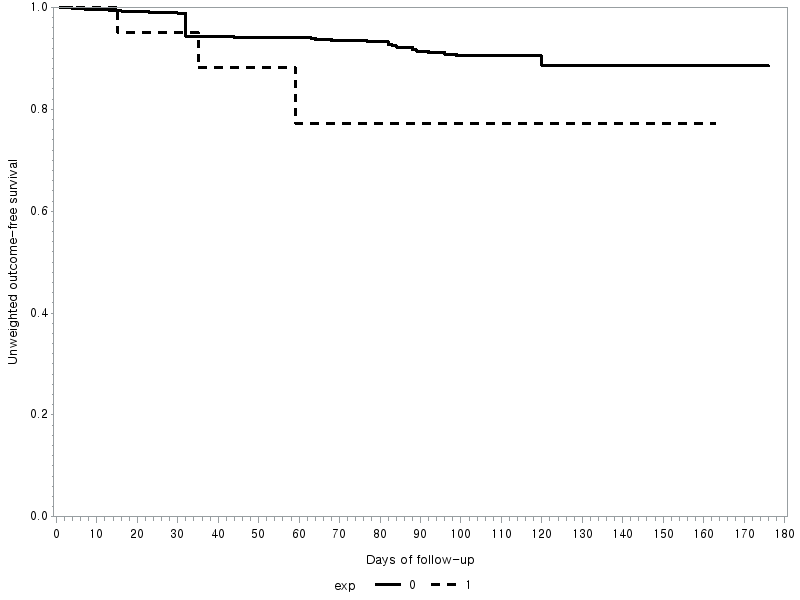   \| Number at risk \| Duration of follow-up (days) \| \| \| \| \| \| \| \| --- \| --- \| --- \| --- \| --- \| --- \| --- \| --- \| \|  \| 0 \| 30 \| 60 \| 90 \| 120 \| 150 \| 180 \| \| Non-exposed \| 2653 \| 1070 \| 488 \| 332 \| 139 \| 29 \| 0 \| \| Exposed \| 24 \| 14 \| 6 \| 6 \| 1 \| 1 \| 0 \| |
|  |
|  |
| 1. VTE |
| 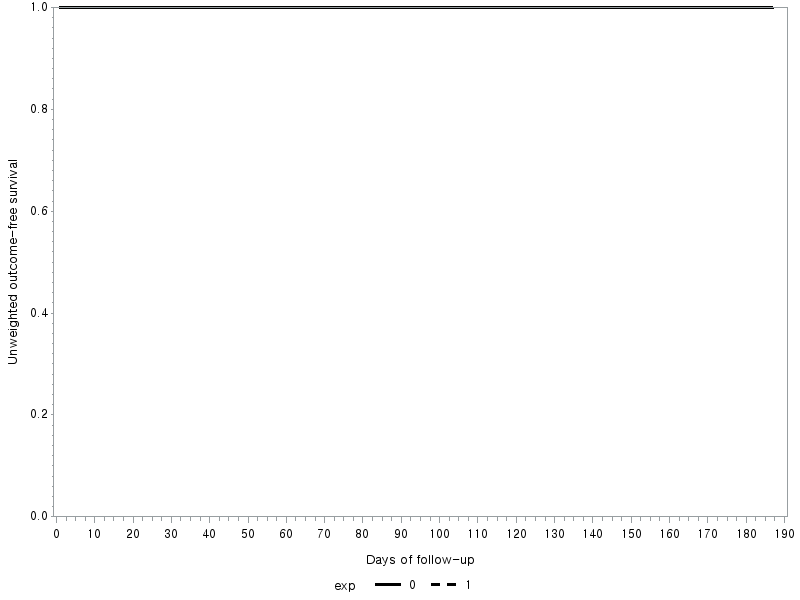   \| Number at risk \| Duration of follow-up (days) \| \| \| \| \| \| \| \| --- \| --- \| --- \| --- \| --- \| --- \| --- \| --- \| \|  \| 0 \| 30 \| 60 \| 90 \| 120 \| 150 \| 180 \| \| Non-exposed \| 2653 \| 1088 \| 550 \| 397 \| 175 \| 39 \| 1 \| \| Exposed \| 24 \| 15 \| 7 \| 6 \| 1 \| 1 \| 0 \| |
| 1. Major bleeding |
| 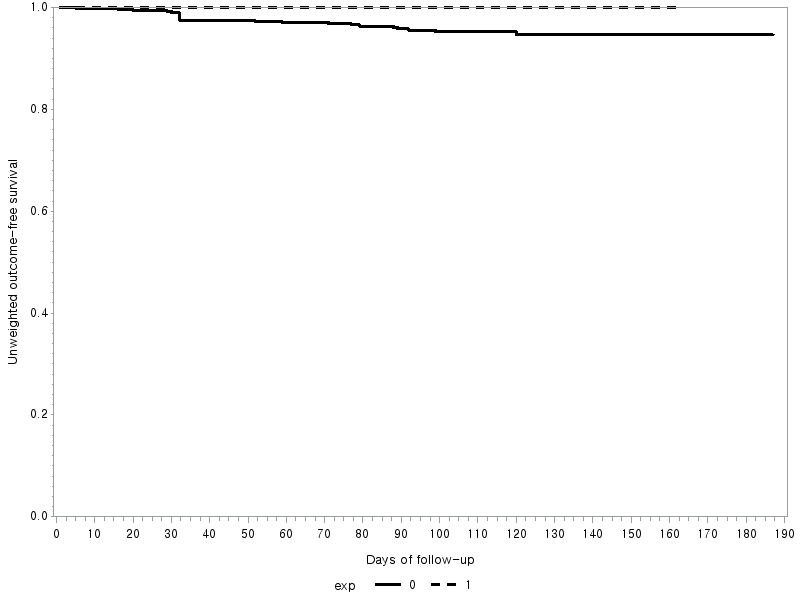   \| Number at risk \| Duration of follow-up (days) \| \| \| \| \| \| \| \| --- \| --- \| --- \| --- \| --- \| --- \| --- \| --- \| \|  \| 0 \| 30 \| 60 \| 90 \| 120 \| 150 \| 180 \| \| Non-exposed \| 2653 \| 1071 \| 519 \| 365 \| 157 \| 36 \| 1 \| \| Exposed \| 24 \| 15 \| 7 \| 6 \| 1 \| 1 \| 0 \| |
|  |
|  |
| 1. ICU admission |
| 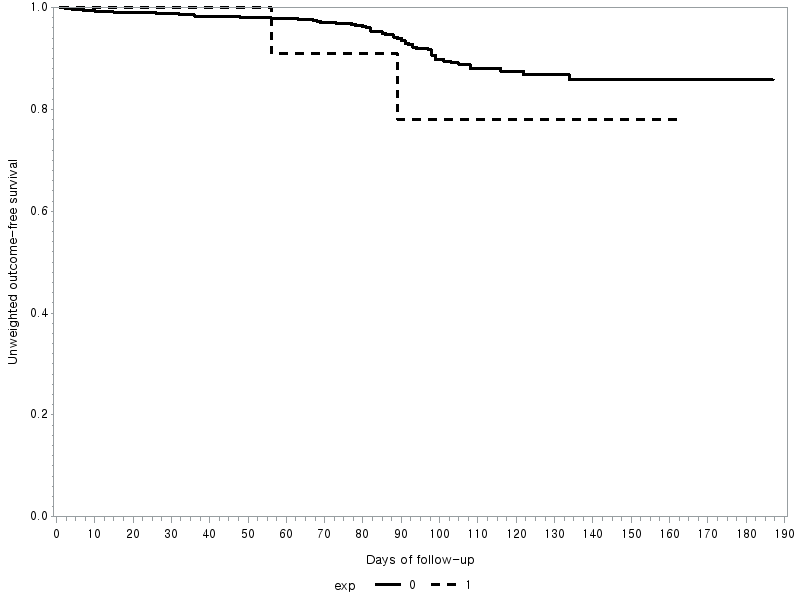   \| Number at risk \| Duration of follow-up (days) \| \| \| \| \| \| \| \| --- \| --- \| --- \| --- \| --- \| --- \| --- \| --- \| \|  \| 0 \| 30 \| 60 \| 90 \| 120 \| 150 \| 180 \| \| Non-exposed \| 2653 \| 1082 \| 550 \| 388 \| 155 \| 33 \| 1 \| \| Exposed \| 24 \| 16 \| 8 \| 5 \| 1 \| 1 \| 0 \| |

**Figure S3.** Distributions of propensity scores for use of anticoagulants at baseline.

| 1. Prior to trimming |
| --- |
| 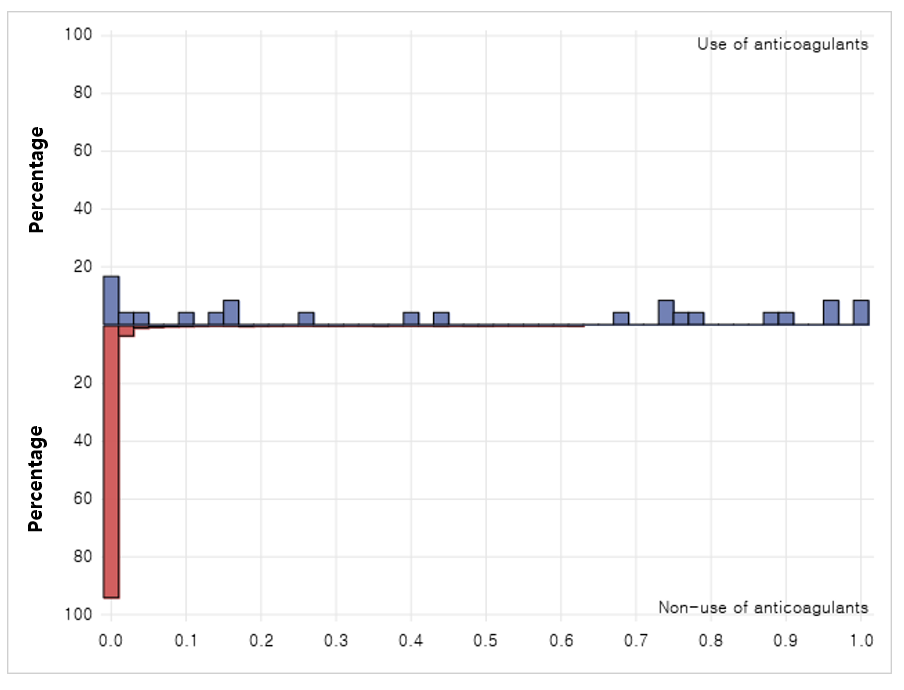 |
| 1. After trimming |
| 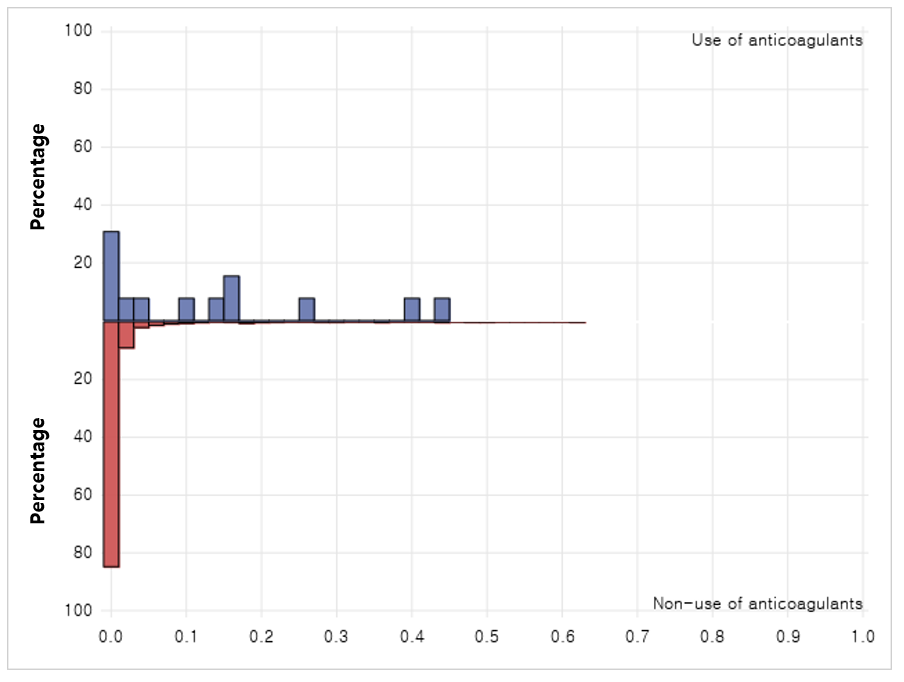   \| Group \| # of trimmed patients \| \| --- \| --- \| \| Non-use of anticoagulants \| 1,627 \| \| Use of anticoagulants \| 11 \| |

*Variables included in the propensity score model: baseline covariates including age, sex, calendar time of cohort entry, type of insurance, numbers of unique medication use, numbers of hospitalization, comorbidities [obesity, hypertension, chronic kidney disease, diabetes, coronary artery disease, cerebrovascular disease, atrial fibrillation, heart failure, stroke, myocardial infarction, mechanical heart valves installation, coronary revascularization, venous thromboembolism, cancers, bleeding], medication use [antidiabetic drugs, antihypertensive drugs, nonsteroidal anti-inflammatory drugs, antiplatelets, statins, immunosuppressants, antivirals, corticosteroids, remdesivir],surgeries
